# Interactions Between Nanoparticles and Dendritic Cells: From the Perspective of Cancer Immunotherapy

**DOI:** 10.3389/fonc.2018.00404

**Published:** 2018-09-25

**Authors:** Jianbo Jia, Yi Zhang, Yan Xin, Cuijuan Jiang, Bing Yan, Shumei Zhai

**Affiliations:** ^1^Key Laboratory for Water Quality and Conservation of the Pearl River Delta, Ministry of Education, Institute of Environmental Research at Greater Bay, Guangzhou University, Guangzhou, China; ^2^School of Chemistry and Chemical Engineering, Shandong University, Jinan, China; ^3^School of Environmental Science and Engineering, Shandong University, Jinan, China

**Keywords:** nanoparticles, physicochemical properties, DC targeting, cancer immunotherapy, DC functions

## Abstract

Dendritic cells (DCs) are the primary antigen-presenting cells and play key roles in the orchestration of the innate and adaptive immune system. Targeting DCs by nanotechnology stands as a promising strategy for cancer immunotherapy. The physicochemical properties of nanoparticles (NPs) influence their interactions with DCs, thus altering the immune outcome of DCs by changing their functions in the processes of maturation, homing, antigen processing and antigen presentation. In this review, we summarize the recent progress in targeting DCs using NPs as a drug delivery carrier in cancer immunotherapy, the recognition of NPs by DCs, and the ways the physicochemical properties of NPs affect DCs' functions. Finally, the molecular pathways in DCs that are affected by NPs are also discussed.

## Introduction

The human body relies on two well-coordinated immune mechanisms for foreign invader defense, antigen non-specific innate immunity and highly evolved antigen specific adaptive immunity. The major functions of innate immunity are to rapidly eliminate pathogens and in a late stage, to transmit risk signals to adaptive immunity to activate the specific responses. While macrophages and natural killer (NK) cells primarily degrade and remove pathogens in non-specific ways, antigen processing cells (APCs) process and present pathogen signals to adaptive effector cells including T cells (1) and B cells (2, 3). Dendritic cells (DCs) are professional APCs. Originating from the bone marrow as progenitor cells with high phagocytic capabilities, DCs undergo maturation in the peripheral lymphatic organs. Maturation of DCs is triggered by pathogen uptake and is characterized by morphological changes, the expression of co-stimulatory molecules and the release of cytokines. During the maturation process, DCs migrate to lymphatic organs, where they activate both memory and naïve T cells, and thus are regarded as the most potent APCs. Because of their central role in inducing adaptive immunity, in recent decades, DCs have been extensively studied toward the aim of vaccine development and cancer immunotherapy (4, 5).

Cancer immunotherapy is regarded as an important progress for cancer treatment in the first decade of the 21st century. The success of some small-scale trials based on two major strategies, checkpoint blockage with antibodies and *ex vivo* T cells engineering, has boosted the development of cancer immunotherapy in recent years (6, 7). Because of the limits of these approaches (8), a third strategy, DC vaccination has been considered (9). Although less developed and with unknown efficacy, some clinical trials based on this approach have shown promise (10, 11). In this strategy, DCs are manipulated by either *ex vivo* or *in vivo* approaches. Compared to an *in vivo* approach in which molecules are directly applied to patients to target DCs, in an *ex vivo* approach, DC precursors isolated from patients are stimulated in the laboratory for maturation by specific antigen and adjuvants, and then are applied back to the patient to activate adaptive immunity. Until now, the functional equality was unknown between *ex vivo* and *in vivo* matured DCs, but studies implicate that DCs undergo maturation differently *ex vivo* than *in vivo*.

**Figure 1:**
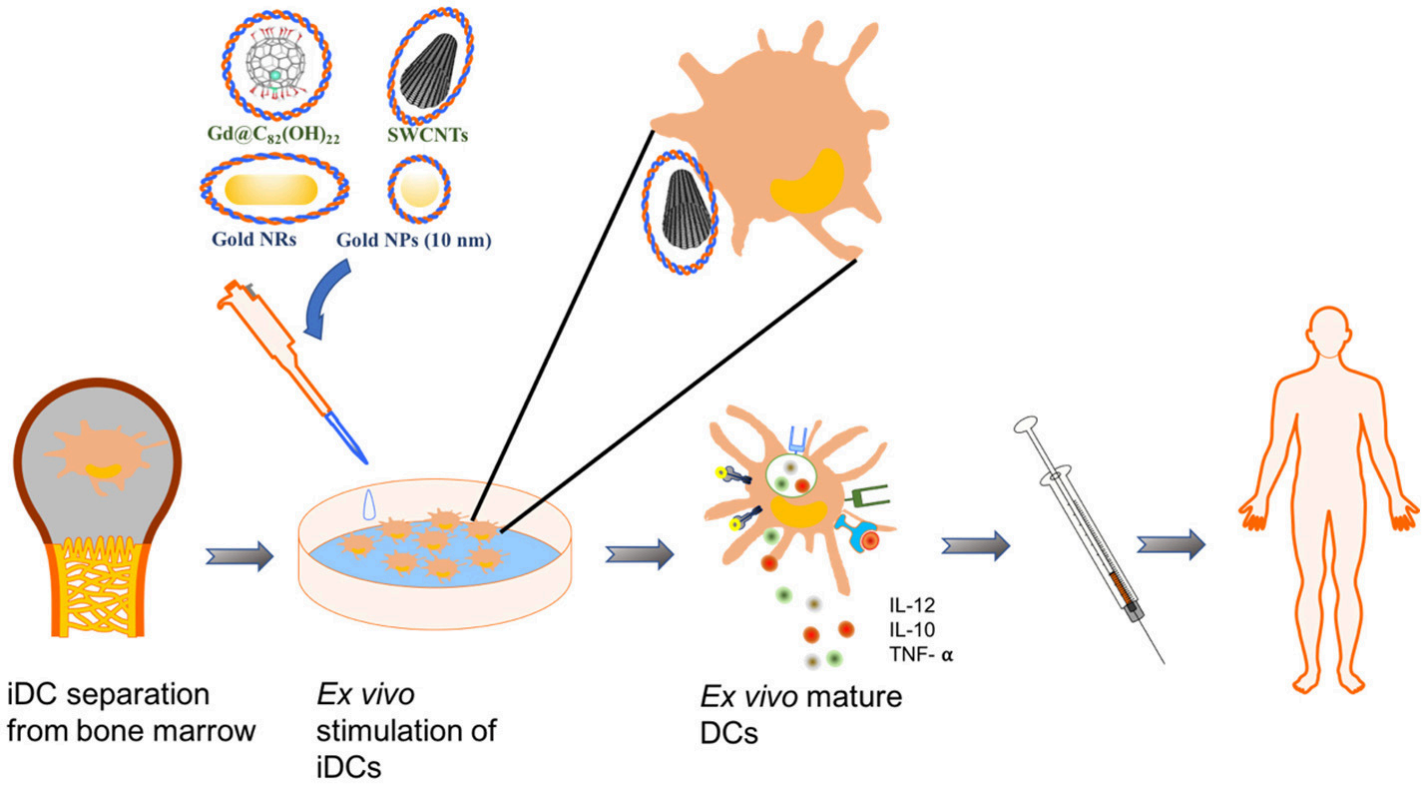
Schematics of an example using NPs for DC-mediated immunotherapy. In this example, immature DCs (iDCs) are harvested from patients' bone marrow. NPs loaded with effect molecules are used to pulse and stimulate the maturation of iDCs *ex vivo*. The mature DCs were reapplied to patients for immunotherapy.

Nanoparticles (NPs) in human blood and lymph are primarily captured by macrophages in the circulation and in tissues composed of the reticuloendothelial system, such as the liver and spleen. The latest studies revealed that precursor DCs patrolling the blood and immature DCs residing in peripheral tissues, such as the kidney and skin, also actively capture NPs, and subject their functions to alteration. These discoveries encouraged interests in using NPs to control DC functions in favor of cancer immunotherapy. The physicochemical attributes of NPs make them especially intriguing for both *ex vivo* and *in vivo* DC manipulation. As a novel DC targeting tool, NPs have at least the following advantages compared to traditional tools. (1) NPs such as gold nanorods (12) and carbon black NPs (13), are adjuvants *per se* and are able to prime DC maturation, thus enhancing humoral and cellular immune responses by synergizing the immunogenicity of antigens. (2) By optimizing the physicochemical properties, NPs can carry antigens or vaccines and directly deliver them to the mature DCs within the secondary lymph organs. In this way, stronger immune responses can be achieved without the accompanying immune tolerance induced by premature DCs (14–16). (3) NPs protect some antigens, such as peptides, from degradation by proteases (17, 18). (4) Using NPs as a platform, co-delivery of two or even more moieties can be realized to achieve stronger immune responses (17–25). Widely used combinations include tumor antigens together with adjuvants such as Toll-like receptor agonists (18, 23–30), or antigens with siRNAs, which silence immunosuppressive genes (31). This property is especially important for some antigens with weak immunogenicity (18). (5) Within DCs, a sustainable release of therapeutics can be achieved by NP carriers through chemical modification on their surface, thus activating DCs more efficiently (22, 32). Until now, different NPs, such as carbon nanotubes (CNTs) (23–25, 33), gold NPs (16, 34, 35), natural NPs [starch (36) and chitosan (37)] or synthetic polymer NPs (38–40) have been studied as vaccine carriers and/or adjuvants by distinct strategies. Even though the rapid advancement, the *status quo* is that this is still a newly emerging field and its maturation has been hindered by hurdles such as safety of NPs, and a costly and time-consuming process to harvest DCs from patients. An example of using NPs to stimulate the *ex vivo* maturation of immature DCs (iDCs) for immunotherapy was shown in Figure 1. NP applications for DC manipulation have been well reviewed in some recent excellent literature (41–43). In this article, we first summarize the latest progress of using NPs for cancer immunotherapy. With the aim of providing mechanistic insights on NPs-DCs interactions, we next focus on the latest knowledge in the recognition and uptake of NPs by DCs, and the ways NPs affect DC functions. We also discuss possible mechanisms underlying these effects.

## DC-targeting strategy using NPs for cancer immunotherapy

By using NPs as a multifunctional drug delivery carrier for *in vivo* DC-targeting, some promising cancer immunotherapeutic outcomes have been achieved. In some cases, animal survival rates improved because of the success of tumor growth inhibition. For example, multi-walled CNTs (MWCNTs) loaded with cancer testis antigen and Toll-like receptor agonist were quickly taken up by DCs after administered to animals. Inside DCs, the carried antigen was slowly released, driving DCs to continually activate CD4^+^ and CD8^+^ T cell immune responses. Consequently, tumor development was greatly delayed and mouse survival was prolonged (23). In another study, co-delivery of OVA and immune-adjuvants by MWCNTs to DCs dramatically inhibited the growth of OVA-expressing melanoma cells in mice (44).

In some other studies, cancer killing molecules and cytotoxic T cells were activated by a NPs mediated targeting strategy. For example, single-walled CNTs (SWCNTs) carrying a peptide tumor antigen to DCs induced specific IgG responses against this antigen in mice, while there were no such responses when mice were challenged with antigen alone (18). When uploaded by upconversion NPs, the injected ovalbumin enhanced the homing capability of DCs to draining lymph nodes in mice, and significantly induced cytotoxic T lymphocytes and the production of cancer killing molecules such as IFN-γ (17). After administration to mice, gold NPs with polyelectrolyte multilayer coatings increased DC activation and antigen presentation and induced a high level of antigen specific CD8^+^ T cell immune response in blood (45). Although in these studies cancer inhibition or animal survival data were not reported, the stimulation of cancer killing cytokines and cytotoxic T cells is an indication of better cancer therapeutic efficacy. Some more examples of NPs that were studied for DC-targeted antigen delivery were summarized in Table 1.

**Table 1:** NPs used for DC-targeted antigen delivery.

**Category**	**NP materials**	**Antigen/pathogen**	**References**
Liposome	Lipid	NY-ESO-1	(46)
	Lipid	OVA	(47)
	Cationic lipid	OVA	(48)
Polymer	PLGA	OVA and monophosphoryl lipid A	(49)
	PLGA	Tetanus toxoid	(50)
	γ-PGA	OVA	(51)
	Poly(propylene sulfide)	OVA	(52)
Inorganic	MWCNTs	OVA, CpG	(44)
	Upconversion NPs	OVA	(17)
	GNPs	Peptide antigen and receptor agonist	(45)
Viruses particle	Adenoviral vectors	TRP2	(53)

*NY-ESO-1,a human tumor antigen that is highly expressed in melanomas; PLGA, poly(lactic-co-glycolic acid); γPGA, poly-γ-glutamic acid; OVA, Ovalbumin; TRP2, tyrosinase-related proteins 2*.

## Recognition and uptake of NPs by DCs

*In vitro* studies using DC models derived from different species (54–56) revealed that, as in other cell types, the uptake of NPs by DCs is either energy dependent or independent (57). Depending on the physicochemical properties of NPs, all four endocytosis pathways are reportedly used by DCs for NPs uptake (Table 2) (52, 60, 63, 64). For example, small sized NPs take a clathrin- and scavenger receptors-dependent pathway (55), while those with diameters larger than 250 nm enter DCs primarily by clathrin independent ways (60, –62).

**Table 2:** Effect of NPs' properties on cellular uptake.

**ID of ENPs**	**Physicochemical properties**	**Cell source**	**Cellular uptake**	**References**
Gold nanoclusters (GNCs)	~2 nm in size, coated with mixture of zwitterionic and carbohydrate ligands	hDCs from blood	Clathrin-, F-actin-, and C-lectin dependent uptake	(54, 58)
Hybrid TiO_2_/para-amino benzoic acid NPs	5–6 nm	hDCs from blood	Macropinocytosis	(59)
GNPs	~6 nm, coated with ordered or random arranged hydrophilic and hydrophobic groups	DC2.4 cells	Ordered structure: energy-independent; Random structure: energy-dependent	(57)
QDs with cadmium/selenide core and a zinc sulfide shell	18 nm, coated with carboxylic acid	Pig MDCs from blood	Clathrin- and scavenger receptor dependent endocytosis	(55)
Poly(propylene sulfide) NPs	45 nm, labeled with Alexa488	BMDCs	Clathrin-mediated endocytosis, and Macropinocytosis	(52)
PLGA NPs	135 nm, untargeted PEGylated surface	BMDCs	Clathrin-mediated endocytosis, caveolin-mediated endocytosis, and macropinocytosis	(56)
Gelatin NPs	245 nm, carrying model drug	BMDCs	Phagocytosis	(60)
PLGA NPs	360 nm, carrying model drug	BMDCs	Phagocytosis	(61)
PLGA NPs	500~600 nm, carrying model drug	hMDCs from blood	Phagocytosis	(62)

*h denotes human; BMDCs, bone marrow-derived DCs; PEG, Polyethylene glycol.

Physicochemical properties of NPs dictate the recognition and uptake process. Recent studies consistently reveal that smaller NPs are easier for DCs to take up. For example, compared to those with a diameter larger than 1 μm, PLGA NPs of 300 nm have the higher uptake efficiency by DCs (64–66). Another example is that polypropylene sulfide NPs with a size of 20 nm accumulated in DCs in the lymph nodes more efficiently than those of 45 and 100 nm, thus inducing more greater immunity (67). Shorter MWCNTs showed better cellular uptake compared to longer ones, and consequently induced more potent immune responses (25, 33). However, there are paradoxical opinions on the relationship between NPs size and their adjuvant activity, since NPs with different material components have their own optimum size for the induction of immune response (68).

Surface chemistry, such as charge and ligand organization pattern, affects NP internalization by DCs. An example is the positive charge on the gold NP surface led to a higher uptake efficiency by human monocyte-derived DCs (69). Early studies showed that the surface hydrophobicity of NPs was correlated with DCs mediated immune effects (70, 71). For example, hydrophobic segments in amphiphilic γ-PGA NPs' surface significantly increased their interactions with DCs and the consequent immune responses (72, 73). Another example is the zwitterionic ligand coated gold NPs (<3 nm in size) had a higher DCs uptake efficiency compared to those coated with PEGylated ligands (58). In one study, DCs took up NPs coated with PEG-3000 in a higher efficiency compared to those coated with shorter or longer PEG chain (74). Phosphatidylserine modification increased the internalization of SWCNTs by DCs compared to pristine ones or those coated with phosphocholine (75). The presence of aromatic structures on the NP surface resulted in the enhanced uptake of negatively charged GNPs and the activation of DCs (76). Not only chemistry type but also structural organization of surface chemical molecules affects the uptake process by DCs. For example, gold NPs coated with organized striations of alternating anionic and hydrophobic groups penetrated the plasma membrane and entered the cytosol, whereas those with randomly organized functional groups of the same composition were mostly trapped in endosomes of DCs (57).

The shape of the NPs also affects the recognition and uptake by DCs. Compared to spherical counterparts of the same size, gold nanorods and peptide nanofibers showed higher uptake efficiency by DCs in regards to the number of internalized NPs per cell (34, 77). Interestingly, only higher uptake of peptide nanofibers led to a stronger adjuvant efficiency, and gold nanorods actually did not. In another study, the uptake efficiency of rod-shaped PEG-based hydrogel NPs by mouse bone marrow-derived DCs was lower than that of disc-shaped NPs with similar volume and dimensions (78).

In *in vivo* models, physicochemical properties of NPs again govern their interaction with DCs. In one study, cationic NPs more readily associated with both CD11b and CD103 DC subtypes in the lung than anionic ones, thus resulting in a higher expression level of *Ccl2* and *Cxc10*, two important chemokines that recruit DCs into the drainage lymph nodes (79). NPs uptake by DCs in administration sites and the accumulation in drainage lymph nodes was reported after administration by different routes (67, 80–84). In some studies, the uptake of NPs by DCs residing in lymph nodes was also detected (67, 80, 85, 86). Detailed studies showed that the locally injected NPs migrate to lymph nodes by two routes, i.e., through direct draining of NPs, or through DCs migration from injection site to lymph nodes, where NPs are re-taken up (86). Studies suggest that depending on the composition, NPs with a diameter less than 200 nm usually take the first route, while larger ones take the second route (27, 67, 85–88).

## NPs affect DC functions

In peripheral organs and in the blood, premature DCs internalize pathogens or foreign antigens and migrate to draining lymphoid tissue, where they undergo the maturation process characterized by morphological changes and increased expression of cytokines required for priming T cell and membrane molecules, such as CD40, CD80, CD86, DEC205, and MHC molecules (89). In the draining lymphoid tissues, mature DCs activate effector T cells (90). Mature DCs strongly stimulate naïve and memory T cells by presenting antigens. Depending on the way DCs are activated, T cells are stimulated by DCs to differentiate into distinct lineages of T helper (Th) cells, primarily including Th1, Th2, and Th17, which lead to cellular immunity, humoral immunity, and tissue inflammation, respectively. Recent studies revealed NPs affect all steps of DCs induced immunity.

### NPs affect DC maturation

Long-term immune protection against tumors or pathogens requires the expansion of antigen-specific effector and memory T cells. Naïve T cell expansion can only be efficiently stimulated by mature DCs; therefore, the maturation of premature DCs is critical for the realization of efficient immunotherapy. Recent studies have shown that NPs favor the maturation process of DCs. For example, in *in vitro* culture, γ-PGA-Phe NPs induced a significant increase in the expression of maturation markers of DCs, and this capability is size-dependent (39, 86, 87). Gold nanorods were also reported to promote DC maturation and downstream immunity, and this effect depended on their surface chemistry (35, 91). Chitosan and other polymer NPs induced DC maturation in a similar way to LPS treatment (92, 93). According to recent research, ZnO NPs at a concentration of 30 μg/mL upregulated the expression of costimulatory molecules CD80 and CD86, and the secretion of IL-6 and TNF-α (94), but they did not show such effects at lower concentrations (10 μg/mL) (95). These effects are shape-independent, since spherical and sheet-shaped ZnO NPs with similar specific surface area showed similar effects (94). C60 fullerenes (96), carbon black (97, 98), (Gd@C_82_(OH)_22_)n (99, 100), and layered double hydroxide (LDH) NPs (101) were all reported to increase the expression of MHC and co-stimulatory molecules on DC surface. In another study, polyanhydrides NPs activated DCs with an efficiency that was dependent on their shape and surface hydrophilicity/hydrophobicity (102).

However, NPs are also reported to inhibit the maturation of DCs. For example, treatment with negatively charged QD655-COOH (18 nm) suppressed the expression of CD80/CD86 stimulated by LPS in porcine monocyte-derived DCs (55). In human DCs, gold NPs with a diameter of 10 nm inhibited the expression of CD86, CD83, and IL-12p70 induced by LPS treatment (103). These findings warrant further investigations to avoid side effects of NPs when used for cancer immunotherapy.

### NPs affect homing capability of DCs

To activate T cells, DCs must migrate to lymphoid organs and localize closely to the residence T cells, a process known as DCs homing. A high homing efficiency is critical for a successful DCs-based immunotherapy. The homing of DCs can be improved by approaches including pre-injection of pro-inflammatory cytokines or DC homing receptors (104), and optimization of DCs' administration route and times (105). Even with this, the DC homing efficiency remains unsatisfactory (106). Recently, magnetic NPs have been used under an external magnetic field to promote the homing capability of DCs after *in vitro* activation (107). Some studies suggested that *in vitro* pulsed DCs by NPs accumulate in draining lymph nodes (17, 18), however, very little is known about NPs' effects on homing capability of DCs. One study suggested that NPs may improve DC homing by increasing the expression of chemokine receptor 7 (CCR7) on DCs surface and leading to the rearrangement of the cytoskeleton (16).

In addition to chemokines on the surface, it is well known that the maturation status of DCs determines their migration to draining lymph nodes (108). It is possible that NPs affects DCs' homing capability by influencing the maturation status of DCs. Future studies are necessary to further understanding of this aspect.

### NPs compromised antigen processing and presentation capability of DCs

After digestion by APC cells, antigen fragments will be displayed on the cell surface together with either class I or class II MHC molecules, and consequently are recognized by CD4^+^ (helper) or CD8^+^ (cytotoxic) T cells, respectively. This process is known as processing and presentation of APC (109–111). It is well reported that NPs change the antigen processing capability of DCs. *In vivo*, after pharyngeal aspiration, SWCNTs inhibited DCs' functions of antigen capture/processing and presentation but not their maturation process, thus causing decreased proliferation of splenic T cells (112). In *in vitro* culture, murine DCs treated with graphene oxide (GO) engulfed antigen normally but showed an impaired capability to process antigen and activate antigen-specific T lymphocytes (96). This effect was specific to GO and was not found for other carbon-based NPs, probably due to its planar and negative charged surface. Another example is that treatment with super-paramagnetic iron oxide NPs (PVA-SPIONs) compromised DCs' capability of processing model antigen DQ-OVA with or without concomitant LPS exposure but did not impair their maturation after antigen uptake. As a consequence, the expression of MHCII and the capacity to stimulate autologous CD4^+^ T cells *in vitro* were compromised (113).

Harnessing the ability of DCs to induce antigen-specific CD8^+^ T cell immunity (cross-presentation) is crucial for development of antitumor vaccines. As potential vaccine carriers, NPs could enhance antigen cross-presentation of DCs, thereby produce stronger antitumor immunity. For example, as shown in both *in vivo* and *in vitro* experiments, treatment with polymer NPs such as γ-PGA NPs (114, 115), PLGA NPs (116), polyethyleneimine NPs (117) and poly(propylene sulfide) NPs (52) promoted OVA-mediated cross-presentation in DCs by different mechanisms. Changing size and surface chemistry of NPs were effective ways to regulate their effects on antigen cross-presentation intensity of DCs (118, 119). Some metal oxide NPs such as aluminum hydroxide (120) and super-paramagnetic iron oxide NPs (SPIONs) (121) were also reported to improve the cross-presentation ability of DCs. These above studies suggested a potential strategy of using nanotechnology to develop DCs-based cancer immunotherapy.

### NPs affect DC induced T cell differentiation

NPs have long been known to affect DCs' induction of T cell differentiation. For example, exposure to carbon black NPs or diesel exhaust of different sizes significantly enhanced the capacity of bone marrow-derived DCs to stimulate T-cell proliferation (97, 98). When cultured with CD4^+^ T cells, human monocyte-derived DCs after treatment with poly(vinylalcohol)-coated SPIONs (PVA-SPIONs) showed an impaired capability to activate CD4^+^ T cell and altered the cytokine release profiles (113). Mechanistically, NP exposure suppressed DCs' capacity to process and present antigen. In another study, porous silicon NPs modified by high C-H structures strongly enhanced DCs' activation of T cell differentiation (122).

Some NPs promoted DCs capability to stimulate both Th1 and Th2 differentiation (123). However, recent studies revealed that treatment with NPs may bias DC induced T cell differentiation directions. For example, SWCNTs (124), (Gd@C_82_(OH)_22_)_n_ (99, 100), magnetic iron oxide NPs (MIONs) (125), oxidative TiO_2_ NPs (126), and PLGA NPs (127) were reported to increase Th1 cell proliferations, while 10 nm gold NPs (103) and CeO_2_ NPs (126) potentiate DCs' capability to promote Th2 polarization. Moreover, this polarization effect is related to the surface chemistry. For example, gold nanorods coated with poly(diallydimethylammonium chloride) (PDDAC) and polyethyleneimine (PEI) showed a Th2 polarization activity, while those coated with cetyltrimethylammonium bromide did not (35). In another study, TiO_2_ NPs induced DC maturation and polarized T cells toward Th1-based responses, while CeO_2_ NPs treated DCs induced Th2-dominated T cell profile (128). Some NPs, such as polystyrene NPs (50 nm) (129), were found to inhibit Th2 polarization without affecting Th1 immunity. Not only NPs composition but also the NPs treatment conditions determines DCs induction effects on T cell differentiation. For example, SWCNTs at 0.5 μg/mL increased Th1 cell proliferation, while suppressing it at 10 μg/mL (124).

NPs were also reported to affect DCs' induction of T cells to differentiate into Th17 cells. For example, carbon black induced Th17-dependent inflammation in mice (130) and gold NPs of 50 nm (but not 10 nm) favored Th17 polarization (103). In comparison, PLGA NP induced nasal tolerance and inhibited T-cell differentiation into Th17 cells (131).

**Figure 2:**
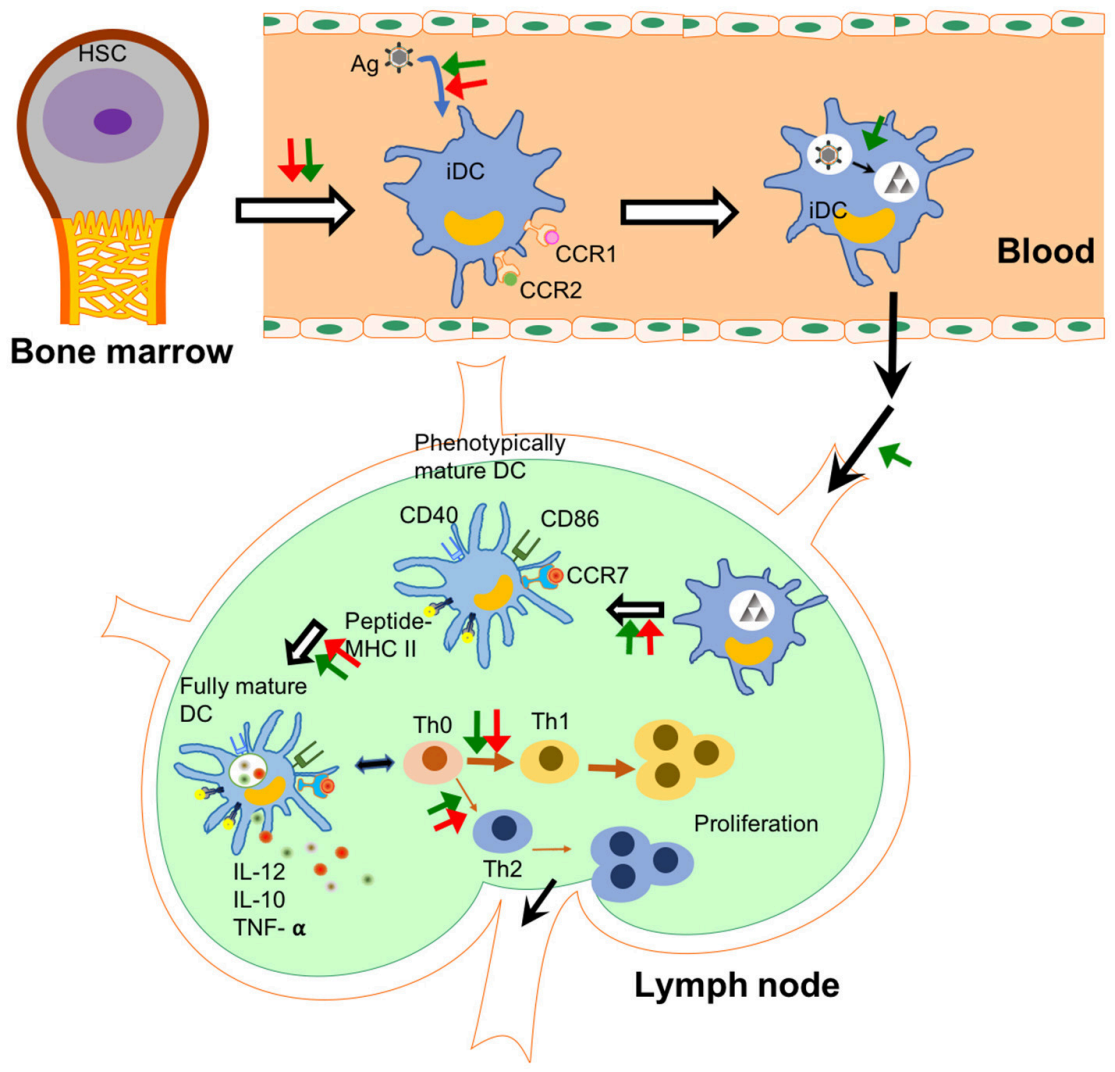
NPs affect DCs' functions in different steps. The checkpoints of DC immunology are shown in this figure and the steps under the probable influence of NPs are summarized. NPs affect the differentiation from haematopoietic stem cells (HSCs) to immature dendritic cells (iDCs) in the bone marrow (132). They change the capability of DCs to uptake and process antigens in peripheral tissues. Some NPs enhance the homing capability of DCs into lymph node. In lymph node, NPs affect antigen presentation capability and maturation of DCs including the release of pro-inflammatory cytokines. Finally, NPs lead to polarization of T cell differentiation induced by DCs. Green arrows show an enhancement effect, while red arrows show an inhibition effect. Black arrows indicate the flow of immune cells.

To sum up, the uptake of NPs affects DCs' functional steps from maturation to induction of T cell differentiation (Figure 2). The NPs' physicochemical properties and exposure scenarios govern the outcomes and intensity of these effects. The interactions between NPs and DCs may favor or impair the functions of DCs in immunotherapy. While some molecular mechanisms have been proposed, more are yet to be revealed.

## Possible mechanisms: molecular pathways in DCs affected by NPs

Understanding the immunomodulatory mechanisms is a premise to optimize the functions of NPs for immunotherapy. Currently available literature supports that the effects of NPs on DCs may be triggered by binding with extracellular membrane receptors or acting on intracellular molecules. Both interactions depend heavily on the physicochemical properties, especially the surface chemistry of NPs.

**Figure 3:**
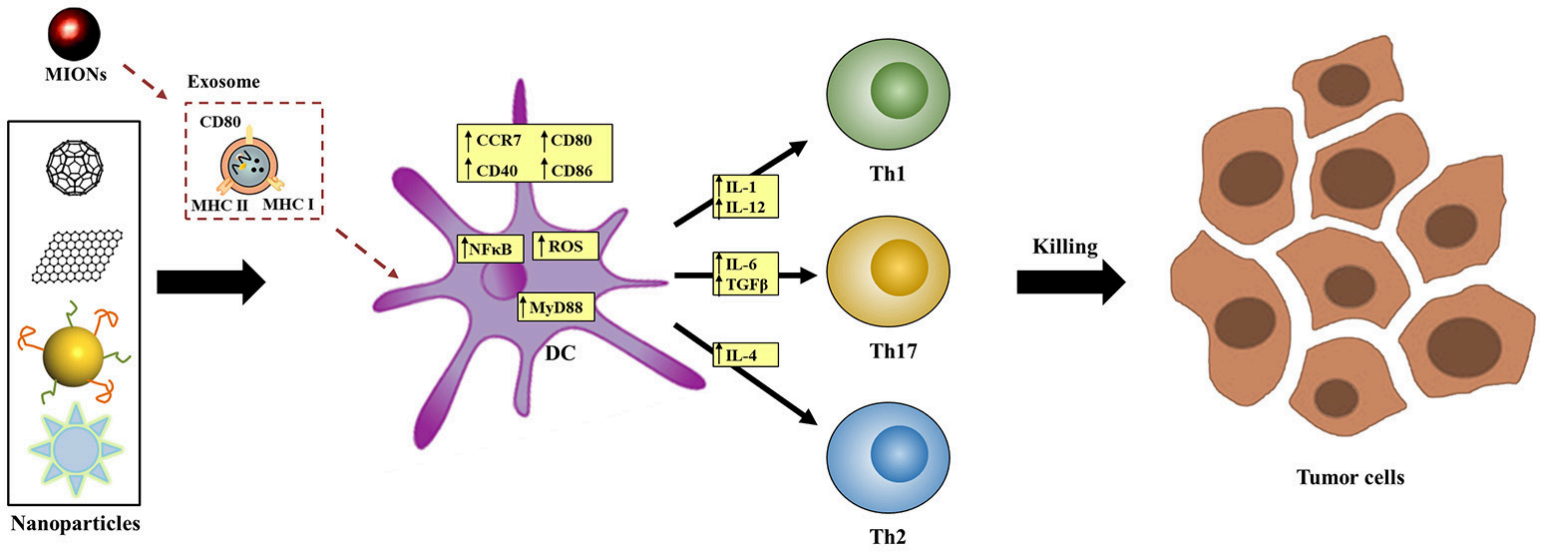
Proposed molecular mechanisms by which NPs affect DCs' functions. NPs may interfere signal transduction (e.g., TLR-MyD88 signaling), exosome-mediated process, intracellular redox balance, or calcium oscillation inside DCs to affect DCs' functional process.

One possible mechanism by which NPs affect DCs' fate is via the interference of the intracellular signaling pathways after recognition by cell surface receptor(s). Recent studies have proposed TLR (Toll-like receptor)-MyD88 signaling (133) as one of the most likely pathways that mediates NPs' effects. This mechanism was first identified by a micro-array analysis (134) and was later supported by observations in MyD88-knockout and TLR4-deficient DC models and mice (39). For example, after treatment with γ-PGA NPs, the maturation of DCs with MyD88- or TLR4-knockout but not wild-type was impaired. In addition, in wild-type mice, NPs augmented OVA-induced adaptive immune responses, including T cell activation and anti-OVA antibody production, but these effects were largely diminished in TLR4-deficient mice (39). In some studies, the activity of NF-κB and MAPK signaling pathways, both of which are downstream of TLR-MyD88 signaling, was found changed by NPs treatment in DCs (135). These results further support that TLR4-MyD88 is at least one of the signaling pathways that mediated the effects of NPs (Figure 3).

Another reported mechanism is related to the generation of exosomes. *In vivo* investigations showed that magnetic iron oxide NPs (MIONs) stimulated the generation of exosomes in the alveolar region of mice after respiratory exposure (125, 136). These exosomes transferred to the reticuloendothelial and immune systems, where the maturation of DCs was induced. *Ex vivo* studies indicated that when incubated with MION-Exo, immature DCs underwent maturation, as shown by the stimulated expression of MHC class II I-A^d^, MHC class I H-2K^d^, CD80, and CD86, and differentiated into DC1 subtype as shown by the increased secretion of cytokine IL-12. In contrast, MIONs *per se* did not have the same effects, suggesting that MIONs-induced exosomes mediated the effects of MIONs in stimulating host immunity (Figure 3).

Some other plausible mechanisms have also been proposed. Based on the link between redox equilibrium with phenotypic and functional maturation of DCs (137), oxidative stress may play a key role for DC activation after treatment with NPs including carbon blacks (CBs) (130, 138) and SWCNTs (124). In one study, 10 nm gold NPs were found to inhibit the change of Ca^2+^ oscillation during LPS-induced DC maturation (103). However, blocking Ca^2+^ oscillation cannot totally impair DCs' maturation, suggesting that calcium oscillations-dependent signaling is not the sole target of gold NPs in the regulation of DCs' maturation.

Till now, our knowledge about the ways NPs modulate intracellular molecular pathways in DCs is very limit. Since the interactions between NPs and intracellular molecules may be dominated by the physicochemical properties, especially the surface chemistry of NPs, future studies of the structure-activity relationship will help rational design of NPs-based tools to harness immunotherapeutic functions of DCs.

## Perspective

For successful DCs-based immunotherapy, three strategies have been considered. First, to deliver tumor specific antigens to DCs and to stimulate their maturation *ex vivo* followed by re-infusion back to patients. Second, *in vivo* targeting of DCs with DC-specific targeting molecules together with tumor antigens and activators to induce cytotoxic T cell activation. Finally, *in vivo* targeting of DCs augments tumor rejection inflammation in the tumor microenvironment (9). For all of these strategies, NPs are perfect antigen or adjuvant delivery carriers of high molecular quantity and variety. *In vivo* manipulation can better mimic the natural maturation process of DCs, thus can probably produce safer and more efficient immunotherapeutic outcomes. In the future, a deeper understanding of the mechanisms by which the physicochemical properties of NPs affect DCs' functions, such as maturation, homing, antigen process, and induction of T cell differentiation, will be required for safe and efficient DCs-based cancer immunotherapy.

## Author contributions

JJ, YZ and SZ designed this work of review. YX and CJ performed the literature search of the databases. JJ, YZ, and SZ wrote the manuscript. YZ and BY revised the manuscript. All authors approved the paper for publication.

### Conflict of interest statement

The authors declare that the research was conducted in the absence of any commercial or financial relationships that could be construed as a potential conflict of interest.
